# Calcification of Dental Pulps

**Published:** 1895-01

**Authors:** A. Rose

**Affiliations:** Petersborough, Ont.


ARTICLE II.

### CALCIFICATION OF DENTAL PULPS.

BY A. ROSE, L. D. S., PETERSBOROUGH, ONT.

The title of this paper as given in the programme would more accurately describe the subject if written, "Calcific deposits in the dental pulp chamber," because these deposits seldom assume the appearance of a calcified pulp.

Calcific deposits, as generally found in a pulp chamber of the human tooth by the dentist in ordinary practice, vary in quantity from a very thin incrustation adhering to the surface of the pulp, to a mass of semitranslucent substance usually resembling dentine in appearance and structure and completely filling the chamber and canal. They often occur in small granular particles or spiculæ

through the pulp tissue, and also either attached to the walls of the chamber or to the sheath of the pulp, as minute pearls ranged along it. Many specimens, when removed from the chamber, appear to be cone-shaped, with the base spread out towards the opening in the dentine produced by the caries. Others show very irregular shapes.

Starting from a base looking towards the approaching caries or other irritating causes, they seem to penetrate the chamber, throwing forward projections in the direction of each canal in the roots of the tooth. Also, I would mention those deposits found in the chambers of the teeth of older persons when abrasion from the work of mastication has resulted in wearing the teeth to the gums, perhaps. In this class the canal seems to be in many cases simply closed up from the deposition of calcific matter upon its walls surrounding the pulp, until the whole cavity is filled and the pulp disappears entirely. As a last class, I would mention what seems to me to resemble more than any other class a real case of calcification of the pulp. These are found in the teeth of very old persons, in which I have found the pulp to assume the appearance of the pith of a goose-quill but to possess a firmer structure, being more like a piece of the quill itself.

In all cases but the last two, those specimens which I bave discovered and now have to exhibit, on being removed from the chamber and canal, were enclosed or surrounded by the pulp or the sheath of the pulp, except at the base where they were perhaps attached to the dentinal wall of the chamber. The sheath seemed to remain intact, even when the deposit had penetrated the canal nearly to the apex of the root, and when inflamed from any cause whatever (which inflammation is the usual cause of the trouble leading to the discovery of the deposit), this sheath is intensely sensitive to approach and is often possessed of a very tenacious vitality, resisting the action of arsenic and cocaine, and requiring several applications to overcome its sensitiveness.

The structure of these deposits seems, according to the opinions of leading histologists and microscopists, as Miller, Black and Iszlai, to differ, sometimes being organized similar to dentine, sometimes resembling cementum and sometimes bony in structure.

It would appear to one less skilled in histological knowledge of these parts of tooth structure, that the particular formation of these deposits depends upon that portion of the odontoblastic cells retaining most vitality and receiving the necessary stimulus which may be either local or systemic in character. The writer is also led to conclude that these deposits are the result of nature's efforts to repair an injury either received or threatened, and that they are really of a physiological character, and that any irritation which may render sensitive this monitor of the dental organization, the pulp, may engage its reparative function and cause, in many instances, a deposition of calcific matter upon the walls (or within them) of its habitation somewhere in the direction of the irritation, and this matter may be organized in the shape of dentine, cementum or bone, the morphological differences between these elements of tooth structure being very slight; and when we remember that they all originate in the cellular matter of the dental papilla, it is not unreasonabla to suppose that the stimulation above referred to may as readily produce the one form of matter as the other.

A strong reason for deeming this peculiar formation of calcific matter in the pulp chamber physiological rather than pathological as to origin, is the fact that the experience of almost every observer of this formation agrees in the statement that it is found most frequently in well-developed, well-nourished and usually plethoric individuals. I have often acted upon this assumption, and on discovering one, or perhaps two, of the canals of a superior molar closed to the finest broach, and having been attacked with caries which penetrated the tooth to the position of the pulp chamber originally, the tooth had, in the words of the patient, "just rotted away and never ached at all," I have cleaned the remaining root canals, if any, and after thorough antisepting with iodoform and eucalyptus oil, or aristol and eucalyptus oil, which is less disagreeable, or perhaps with hydrarg. bi-chor. 1-500 or 1-1,000, have dried and filled just as I should have filled any ordinary case. I have several such under observation which have been thus dealt with, one two years ago, and three or four for a shorter time, all giving good satisfaction. I feel quite well satisfied myself that the real office of the ordinary "nerve stone," nerve nodule," "pulp stone," "odontome," or "endodonthele," by whatsoever term we may choose to call it, *is not to cause* the intense pain of neuralgia, or pericementitis, or any of those terribly painful conditions which usually procede its discovery, but its work *is to prevent* this trouble, and I would just here ask, "Who knows how often this latter purpose is fulfilled to the letter?" A good idea may be formed of a proper answer to this question by filing down a hundred of those diseased teeth which have been extracted for replacement with artificial dentures, as is done by the student of the first or second year where the system of Dr. Black has been adopted in teaching "*Operative Technique*."

Very many of these cases "never ache but just rot away," and if examined will be found to possess ample evidence of this purpose in the formation of calcific deposits in the pulp chamber.

The diagnosis of this irregularity of tooth structure is comparatively easy where the tooth has been penetrated by caries to the locality of the pulp chamber. Any effort to remove the pulp will expose the presence of the deposit; but where this deposit exists in an apparently sound tooth, and having been produced through some irritation, perhaps of a systemic character, such as often causes loss of enamel at the gingival margin or very great sensitiveness in the same locality, its presence is suggested by extremely painful spasms, or occasional sharp piercing pain in the tooth, or perhaps a condition resembling pericementitis, which can best be ended by making forcible entrance with a good sharp bur, or a sharpened glyddon drill shank, dipped in cocaine crystals and carbolic acid or glycerine frequently while operating, and forcibly removing both pulp and the deposit. The pain above referred to often yields to cold applications, but requires several applications of arsenious acid and cocaine or morphine to effectually end the trouble when access can be got to within even a short distance of the remaining portion of the pulp.

If these few lines result in a thorough discussion of this subject, and in bringing out the experience of our senior members with this *knotty* subject, I shall have accomplished all I expected while trying to express my own. I never flattered myself with the thought that I had gained more knowledge of this subject than any ordinary practitioner of four or five years' service should have acquired, nor in fact quite so much; but, on being asked if I would give a paper, I consented to do so, feeling it the duty of every member of our profession to aid this society as far as he is able in drawing forth what information can be gained from even an imperfect presentation of his views on any subject, and this subject being lately brought to my mind I adopted it for the occasion.—*Dominion Dental Journal.*

